# Enhancing Postharvest Quality of Blackberries: Impact of Sonicated and Microwave-Assisted Pasteurized Edible Coating Gels at Different Storage Temperatures

**DOI:** 10.3390/gels11040243

**Published:** 2025-03-26

**Authors:** Muhammad Nadeem, KeAndre Leaks, Ahmed Abdullah, Julia Sage Adamson Felix, Muhammad Adnan Shahid

**Affiliations:** 1 Horticultural Science Department, North Florida Research and Education Center, University of Florida-IFAS, Quincy, FL 32351, USA; nadeem.muhammad@ufl.edu (M.N.); leaks.k@ufl.edu (K.L.); ahmedabdullah@ufl.edu (A.A.); js.adamsonfelix@ufl.edu (J.S.A.F.); 2 Institute of Food Science and Nutrition, University of Sargodha, Sargodha 40100, Pakistan

**Keywords:** sonication, microwave, edible gels, nano-emulsion, postharvest quality, blackberries

## Abstract

Blackberries (*Rubus fructicosus* L.) are categorized as functional foods, as they are rich in bioactive compounds. Due to limited shelf life and susceptibility to postharvest quality deterioration, it is imperative to investigate postharvest interventions that can prolong the fruit’s quality. This research aimed to develop sonicated and microwave-assisted pasteurized (SMAP) edible gels with citrus peel essential oil (CPEO). Additionally, we aimed to evaluate the effects of different temperatures (4, 20 and 30 °C) on the postharvest quality of the following blackberry treatments:control (C), blanched (B), coated (SMAP) and blanched + coated (B+SMAP). The synergistic effect of B+SMAP coating gels was more effective at maintaining the quality of blackberries after 21 days in storage by inhibiting fruit weight loss by 18% and fruit decay by 65% compared to the control group at 4 °C. The SMAP-coated fruits limited total flavonoid reduction by 23% and total flavanols by 24% when stored at 4 °C after 21 days. The B+SMAP treatment hindered the loss of total phenolic content by 16%, total antioxidant activity by 27% and DPPH radical scavenging activity by 19% under storage at 4 °C for 21 days. We concluded that the SMAP coating gel is an innovative and health-friendly approach for extending the postharvest quality of blackberries during storage.

## 1. Introduction

Blackberries are among the most extensively distributed berries in the Rubus genus (Rosaceae), with species found worldwide [1]. Blackberries (*Rubus fructicosus* L.) are classified as a functional fruit, rich in a variety of bioactive compounds, including polyphenols, vitamins, organic acids and minerals, and they exhibit significant antioxidant properties [2]. The antioxidant properties of blackberries help the body fight against many chronic diseases like cardiovascular diseases, cancer and infectious diseases [3]. Blackberries are naturally low in cholesterol, saturated lipids, sodium and calories. They contain only trace quantities of vitamin A, B and C, and their fibrous structures contain significant nutritional value. Blackberries contain around 4–6 g of fiber/100 g [4]. It has been discovered that they possess protective properties against colon cancer and cardiac disease due to their high fiber content [5].

Blackberries are processed into numerous products as well as consumed fresh because of their delightful aroma and flavor [6]. Nevertheless, postharvest loss of blackberries is a major challenge in the current scenario [7]. Stakeholder profitability and consumer preference are adversely affected by discoloration, leakage and weight loss. Additionally, blackberry fruits with high respiration and transpiration rates may cause organoleptic losses [8]. It is observed that the deterioration of blackberries through mold accounts for up to 42% of losses during storage. Even in the presence of favorable conditions, the storage period does not exceed more than one week [9]. Therefore, preharvest and postharvest practices that enhance the postharvest quality of the fruits have recently become increasingly significant [10].

Berries are also classified as perishable fruits and are highly susceptible to microbial contamination, which can compromise their nutritional value and alter their quality characteristics [11]. Additionally, the potential presence of pathogens (e.g., *Escherichia coli*) and their toxins can pose a risk to the safety of consumers and contribute to foodborne illnesses. While synthetic fungicides effectively combat fruit pathogens, concerns about their impact on human health and the environment drive researchers to explore natural alternatives to prevent microbial growth and spoilage and maintain fruit quality during storage [12,13].

The applications of natural and organic compounds in food have become increasingly significant due to their health benefits, cost-effectiveness and environmental advantages over synthetic non-organic compounds [14]. In this context, plant-derived natural antimycotics have emerged as the optimal substitutes for synthetic chemical preservatives in the enhancement of food quality and safety [15]. These compounds play an important role in the preservation of food by extending the shelf life and ensuring the safety of food products. Preventing the proliferation of pathogenic microbes ensures food safety, while the antioxidant activity in food products plays a key role in extending shelf life by reducing enzymatic activity [16]. Essential oils’ efficacy originates from phenolic compounds, which are a natural and healthy alternative to synthetic preservatives and additives [17,18].

Citrus essential oils (CEOs) have garnered more attention among plant essential oils due to their peculiar fragrance and flavor as well as their broad-spectrum insecticidal, antibacterial and antifungal properties. CEOs prevent microbial proliferation at low concentrations and improve the sensory qualities of fruits [17,18]. Additionally, CEOs are non-toxic and classified as generally recognized as safe (GRAS) for food consumption in the Code of Federal Regulations. The use of essential oils in edible films has gained popularity for their antioxidant, antimicrobial and physicochemical properties, which help reduce enzymatic browning, texture loss and off-flavors [19]. Carboxy methyl cellulose (CMC) is a linear, soluble anionic polysaccharide that is generated from cellulose. It is a natural substance that is used to create hydrogels, films and carriers for active ingredients. CMC is a copolymer that exhibits the ability to engage in both hydrophilic and hydrophobic reactions with different biopolymers and active chemicals [20].

Ultrasonication effectively reduces emulsion particle size by generating and breaking small gas bubbles through mechanical vibration, resulting in homogenized nano-emulsions [21]. Sonication enhances the production of refined nano-emulsions by using acoustic cavitation to rupture droplets and break them up [22]. Sonication plays a role in stability and can cause functional changes in rheological performances and emulsification [23]. This interaction plays a significant role in enhancing the structural integrity, textural attributes and overall stability of nano-emulsions because of their thickening and gelling activity or their surface qualities [24]. It has been reported that nano-emulsions allow for greater biological activity of essential oils due to an increased surface area, which enables the use of lower dosages of essential oil [25]. The antimicrobial and antibacterial efficacy of nano-emulsions against food pathogens can be improved by decreasing the particle size [26].

Microwaves, employed in ovens for processing, are basically non-ionizing radiation that does not possess the ability to break covalent bonds [27]. Microwaves may induce changes due to the arrangement of constituents (water, ions, salts, etc.) to adjust their dipole with that of the microwaves. This results in friction among constituents which leads to an increase in viscosity [28]. In most emulsions, water, the major polar constituent, absorbs microwaves, disrupting hydrogen bonds and generating heat [29].

Thermal processing is commonly used to preserve fruit and vegetable quality and safety [30]. Blanching is a typical food processing technology that denatures enzymes [31], thereby preventing the deterioration of fruits and vegetables. The most frequent method of blanching is using hot water at 70–95 °C for up to 10 min. It is commonly used as a pre-treatment to improve product quality via the inactivation of enzymes [32]. Plant enzymes such as glycosidases, polyphenol oxidases and peroxidases break down antioxidant substances [31]. Blanching reduces browning and quality loss in produce, making it an important part of food processing [33]. Blanching lowers enzyme activity which causes quality degradation [34]. Shorter blanching times diminish enzyme activity. Blanching raised the total phenolic and total flavonoid content of samnamul but decreased the content over time [35]. An optimal blanching duration is crucial for retaining the nutritious and health-promoting properties of fruits and vegetables [36].

Blackberries, being highly perishable, are prone to rapid spoilage due to microbial contamination, physical damage and enzymatic activities. These issues contribute to significant postharvest losses, affecting both economic value and consumer satisfaction. Since traditional methods of preservation, such as synthetic fungicides, often raise concerns regarding their safety and environmental impact, researchers are motivated to seek alternative and more sustainable solutions. Edible coatings are a method commonly used to improve fruit quality and shelf life. They are composed of a thin edible material which coats the skin of the fruit [13]. These coatings extend the postharvest life of fruits while not adding unfavorable properties as they become part of the product. Edible coatings are safe to consume and environmentally friendly [20].

This project explores the potential of SMAP edible coating gels, incorporating citrus peel essential oil, as an innovative and sustainable approach to extend the shelf life and improve the quality of blackberries during storage. By enhancing the physical, chemical and antimicrobial properties of the fruit, these coating gels can effectively reduce microbial growth, prevent moisture loss and slow down enzymatic browning [12,13,18,23]. Additionally, this study investigates the impact of storage temperatures on the effectiveness of these coating gels, addressing the challenges of maintaining blackberry quality under various conditions. This research is essential for developing sustainable and effective postharvest management strategies by reducing waste and improving the quality of blackberries throughout their shelf life. This overall benefits both consumers and producers.

## 2. Results and Discussion

After sorting and washing, the blackberries were divided into four groups, each containing 100 fully mature and uniformly sized fruits. Afterwards, the fruits were subjected to the following treatments: C (control without coating or blanching), B (blanched), SMAP (coated with the SMAP gel), B+SMAP (blanched and coated with the SMAP gel). All of the treated and control fruits were kept at 4, 20 and 30 °C for 21, 5 and 3 days for different fruit quality analyses. The samples for physicochemical analysis were prepared as follows: approximately 10 g of blackberry flesh was homogenized with distilled water (100 mL) and centrifuged (Jouan C312 benchtop centrifuge, Saint Herblain, France) at 4500 rpm for 30 min, followed by filtration through Whatman #1 filter paper.

### 2.1. Effect of Sonicated Edible Coating Gel on Weight Loss (%)

**Figure 1:**
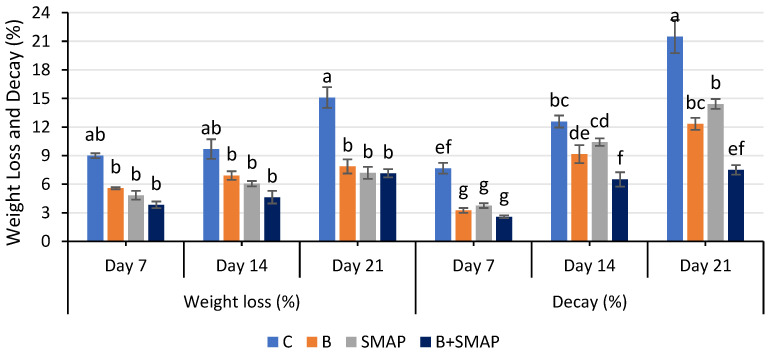
Weight loss (%) and decay (%) of different treatments of blackberries stored at 4 °C. C (control without coating gel), B (blanched), SMAP (coated with SMAP coating gel), B+SMAP (blanched and coated with SMAP coating gel). Different letters show significant differences among treatments at *p* ≤ 0.050.

**Figure 2:**
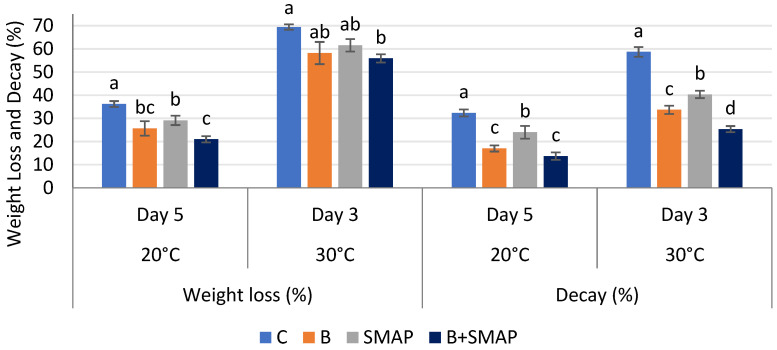
Weight loss (%) and decay (%) of different treatments of blackberries stored at 20 and 30 °C. C (control without coating gel), B (blanched), SMAP (coated with SMAP coating gel), B+SMAP (blanched and coated with SMAP coating gel). Different alphabets show significant difference among treatments at *p* ≤ 0.050.

Weight loss in all of the treatments gradually decreased at 4, 20 and 30 °C as the storage period progressed, regardless of whether the fruits were treated or untreated. At 4 °C after 21 days, the B+SMAP-treated fruits showed a considerable reduction (18%) in weight loss compared to the control fruits (Figure 1). At 20 °C for 5 days, the B+SMAP treatment effectively reduced weight loss by 15% compared to the control (Figure 2). At 30 °C, blackberries treated with the B+SMAP coating gel showed a reduced weight loss of 13% compared to the control after 3 days (Figure 2). So, the effect of the SMAP edible coating gel was more pronounced when it was applied to blanched fruits.

The sonicated (S) and microwave-assisted pasteurized (MAP) edible coating gels proved to be efficient formulations. Additionally, they demonstrated a considerable ability to inhibit fruit weight loss during storage. Weight loss is a crucial aspect that impacts the shelf life of fruit. The effect of the SMAP edible coating gel on the quality of blackberries during storage at various temperatures has yet to be studied. Blanching improves the texture of the blackberries and thus improves their water retention capacity. Increased firmness through blanching blackberry fruit may contribute to maintaining the optimum fruit weight. Further, sonication reduced the particle size of the emulsion, and the MAP increased the viscosity of the gel, which had a more positive impact on reducing water loss and maintaining fruit weight. The findings of this study indicate that the application of the SMAP edible coating gel inhibited a reduction in fruit weight loss during storage. This hindrance can be attributed to the gel’s ability to function as a semi-permeable barrier, effectively preventing moisture loss [37]. Physiological weight loss is a prominent determinant influencing the storage life of fresh food. This phenomenon mostly pertains to the respiration and transpiration processes and water evaporation occurring on the fruit’s surface, potentially resulting in desiccation and the degradation of quality [38]. These processes contribute to the development of undesirable characteristics in fruit, such as wrinkling, diminished brightness and tissue softening [38]. An edible coating containing aloe vera gel inhibited fruit weight loss in Andean blackberries due to the formation of a fine layer over the surface of the fruits [39]. Similarly, an edible cassava starch coating formed a protective layer around strawberries, reducing fruit weight loss during storage by limiting the transpiration and evaporation of moisture from the fruit’s surface [40]. Chitosan and a lactic-acid-based coating emulsion also formed a thin barrier around blackberry fruits, decreasing respiration and moisture loss [41]. In addition to restraining water loss, edible coatings are expected to be semi-permeable to allow gases that are required in processes such as respiration and photosynthesis to pass through [42].

### 2.2. Effect of Sonicated Edible Coating Gel on Decay (%)

The decay percentage increased gradually during storage at 4, 20 and 30 °C. After 21 days at 4 °C, blackberries treated with the B+SMAP treatment indicated a 65% reduction in fruit decay (Figure 1). The B+SMAP treatment caused an 18% inhibition in fruit decay at 20 °C for 5 days and 33% less fruit decay at 30 °C after 3 days. This indicates the effectiveness of blanching in combination with the SMAP coating gel in preserving the fruits from decay.

Citrus peel essential oil (CPEO) possesses antioxidant, anticancer, anti-inflammatory and broad-spectrum antimicrobial properties due to the presence of bioactive compounds. Thus, it is potentially an excellent active ingredient for food safety, antimicrobial packaging and preservatives [18]. Additionally, CPEO can be utilized in food applications through nano-emulsions as it is economical, non-toxic, environment friendly and generally recognized as safe (GRAS) by the United States Food and Drug Administration (USFDA) [43,44,45]. Edible coatings are basically active packaging and when used with CPEO, they provide a maximum protective effect by minimizing fungal growth and extending the shelf stability of food [43,45,46,47]. The protective effect of CPEO is enhanced in nano-emulsion-based edible coatings, as their synergistic action as nanocarriers alters the metabolic processes of fruit. Further, the encapsulated CPEO fragments effectively interact with membranes of microorganisms [16,48,49,50,51,52]. In addition, blanching upholds the structural integrity of cell walls and preserves the firmness of fruit tissue, thereby leading to a decrease in fruit deterioration [53]. Edible coatings exhibit antimicrobial activities that protect fruits from pathogens capable of taking advantage of compromised fruit tissue during storage [54]. This in turn minimizes postharvest losses and equally helps to increase the shelf life of fruits.

### 2.3. Effect of Sonicated Edible Coating Gel on pH

**Figure 3:**
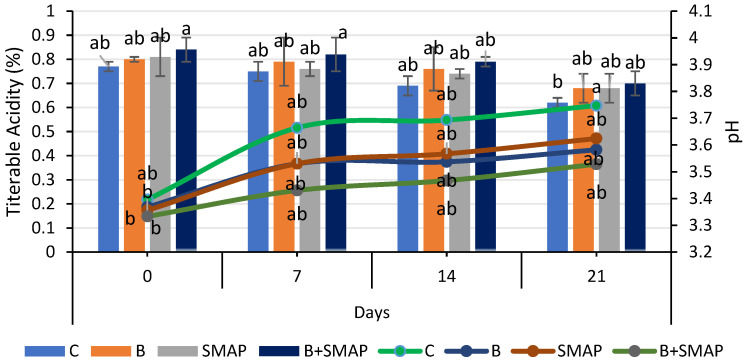
Titratable acidity (%) and pH of different treatments of blackberries stored at 4 °C. Titratable acidity is shown on primary vertical axis (bars) and pH on secondary vertical axis (line). C (control without coating gel), B (blanched), SMAP (coated with SMAP coating gel), B+SMAP (blanched and coated with SMAP coating gel). Different alphabets show significant difference among treatments at *p* ≤ 0.050.

**Figure 4:**
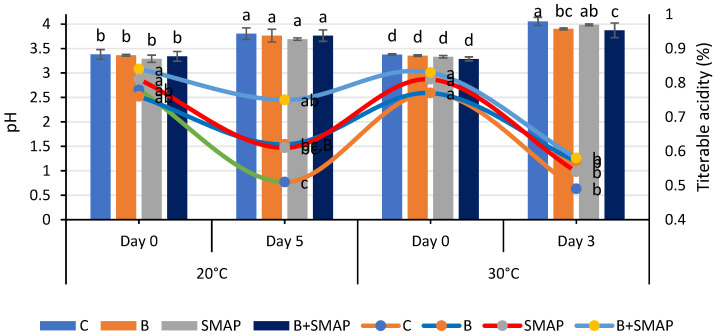
pH and titratable acidity (%) of different treatments of blackberries stored at 20 °C for 5 days and 30 °C for 3 days. pH is shown on primary vertical axis (bars) and titratable acidity on secondary vertical axis (line). C (control without coating gel), B (blanched), SMAP (coated with SMAP coating gel), B+SMAP (blanched and coated with SMAP coating gel). Different alphabets show significant difference among treatments at *p* ≤ 0.050.

Blackberries stored at 4, 20 and 30 °C showed a gradual increase in pH. At 4 °C for 21 days, the minimum increase in pH (5%) was observed in blackberries treated with B+SMAP (Figure 3). At 20 °C after 5 days, the B+SMAP treatment exhibited a comparatively lower increase in pH than the other treatments did. At 30 °C for 3 days, the highest pH (4.05) was recorded in the control blackberries, whereas the lowest was observed in blackberries treated with B+SMAP (Figure 4). So, the synergistic effect of B and the SMAP edible coating significantly limited the increase in pH in different storage conditions.

The pH of fruit is primarily influenced by the presence of organic acids, which are consumed during respiration. As storage progresses, this consumption leads to decreased acidity and an increase in pH [55]. The observed results indicated that the treated fruit demonstrated a consistent pH level in comparison to the control fruit. This can be attributed to the deceleration of metabolic processes as a result of the application of the SMAP edible coating. Our findings align with the results reported by De Bruno et al. [56], who also noticed an increasing trend in the pH of coated strawberries enriched with bergamot essential oil. The findings presented here are also correlated with the results reported by Gol et al. [57], who observed that a rise in pH was greater in uncoated samples compared to the coated strawberries enriched with chitosan. Furthermore, this trend may be attributed to the consumption of organic acids throughout the process of fruit maturation. The alterations in the pH level may be linked to physiological and biochemical modifications occurring throughout the process of respiration [58,59]. The increase in pH during fruit maturation is a result of decreased acidity [60]. The effects of guar-gum- and candelilla-wax-based edible coatings on the properties of blackberries were evaluated during storage. It was observed that pH increased by 8% from 3.60 to 3.90 in coated fruits, while there was a 47% increase, i.e., from 2.60 to 3.83, in uncoated fruits during storage. Although both the coated and uncoated fruits showed an increase in pH during storage, the application of an edible coating enhanced fruit preservation [61].

### 2.4. Effect of Sonicated Edible Coating Gel on Titratable Acidity (TA)

Titratable acidity reduced during storage at 4, 20 and 30 °C. The highest TA (0.70%) was observed in the blanched blackberries coated with the SMAP gel at 4 °C after 21 days of storage (Figure 3). At 20 °C for 5 days, the B+SMAP-treated blackberries exhibited a smaller decrease (10%) in TA compared to the control fruit with a 34% decrease (Figure 4). Similarly, at 30 °C for 3 days, the control fruits experienced a greater loss (36%) in TA compared to the treated blackberries. Although all treatments showed a decline in TA, the B+SMAP treatment helped the blackberries maintain a high TA.

Our results were in accordance with the findings of Rodriguez et al. [62], who also found a decreasing trend in TA in blackberries coated with chitosan and cassava starch stored at 4 °C for 16 days. This decreasing trend in TA in response to the B+SMAP treatment might be associated with physiological processes resulting from oxidation [63]. The decrease in TA during storage may also be due to mold growth on the fruit, with greater deterioration leading to a higher loss of titratable acidity. It is also reported that the TA of the fruits exhibited a steady decline when subjected to greater concentrations of chitosan, CMC + carrageenan and gum arabic, respectively [59,64]. These findings in coated fruits may be attributed to a decrease in respiration rate potentially caused by the application of the coating, which delays the consumption of organic acids. The coatings can alter the exchange rates of gases, thus decreasing both respiration and metabolism [65]. The use of edible coatings, such as guar gum and candelilla wax, effectively helped to preserve the titratable acidity of fruits during storage. It was observed that coated fruits demonstrated higher titratable acidity levels compared to uncoated fruits, with values reaching 1.74% [61]. The delayed degradation of organic acids due to the use of edible coating on fruits plays a crucial role in slowing down metabolic processes and gas exchange [66].

### 2.5. Effect of Sonicated Edible Coating Gel on TSS

**Figure 5:**
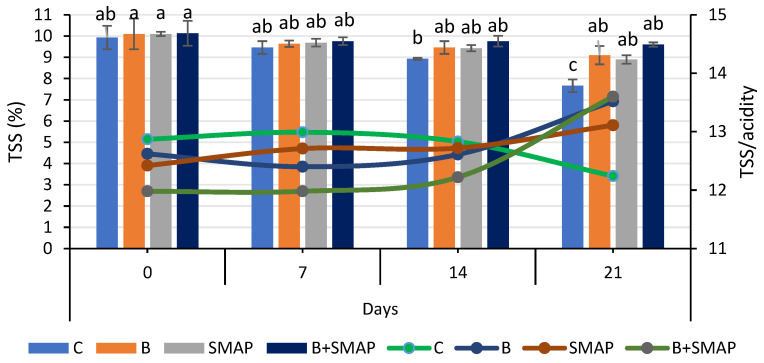
TSS (%) and TSS/acidity of different treatments of blackberries stored at 4 °C. TSS are shown on primary vertical axis (bars) and TSS/acidity on secondary vertical axis (line). C (control without coating gel), B (blanched), SMAP (coated with SMAP coating gel), B+SMAP (blanched and coated with SMAP coating gel). Different alphabets show significant difference among treatments at *p* ≤ 0.05.

**Figure 6:**
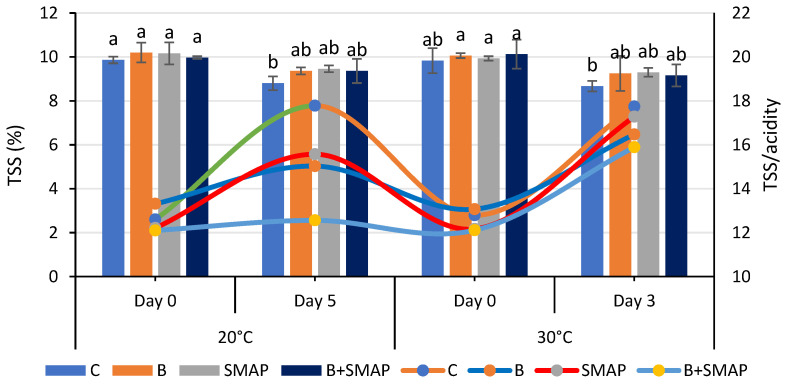
TSS (%) and TSS/acidity of different treatments of blackberries stored at 20 and 30 °C. TSS are shown on primary vertical axis (bars) and TSS/acidity on secondary vertical axis (line). C (control without coating gel), B (blanched), SMAP (coated with SMAP coating gel), B+SMAP (blanched and coated with SMAP coating gel). Different alphbetes show significant difference among treatments at *p* ≤ 0.050.

Total soluble solids were reduced when blackberry fruits were stored at different temperatures (4, 20 and 30 °C) for specific intervals. Fruits treated with B and the SMAP edible coating gel maintained a higher level of TSS during storage. At 4 °C after 21 days, the B+SMAP-treated fruits showed a 13% reduction in TSS (Figure 5). At 20 °C for 5 days, fruits treated with B+SMAP showed a 4% smaller decline in TSS compared to the other treatments. In the case of the 30 °C storage condition, the B+SMAP-treated fruits had the smallest reduction in TSS of 5% compared to the control fruits (Figure 6).

The flavor and nutritional properties of fruit can be assessed by measuring the TSS content. Sugar is the primary component of soluble solids found in the majority of mature fruits. The combination of blanching and the SMAP coating maintained the physicochemical properties of the blackberries. Our results were concurrent with the findings of Oliveira et al. [67], who observed a decreasing trend in the TSS of blackberries treated with an edible coating and stored at 4 °C. The decrease in TSS during storage might be linked to the respiration and the oxidation of the fruit. The variation in TSS in treated and uncoated blackberries was attributed to the physiological processes of the fruit and mold growth during storage, which consequently caused the degradation of sugar [68]. However, this degradation of sugars was slow in coated fruits because coating reduced the rate of respiration and metabolic processes of the fruits by acting as a barrier for gases [69].

### 2.6. Effect of Sonicated Edible Coating Gel on TSS/TA

Blackberry fruits treated with B and/or the SMAP edible coating gel showed an increase in TSS/TA, while the non-treated fruits did not. The lowest (12%) TSS/TA was observed in the control treatment after 21 days; however, in treated blackberries, this ratio gradually increased during storage, reaching its maximum value of 13% after 21 days at 4 °C (Figure 5). Likewise, fruits stored at 20 °C for 5 days showed the greatest (17%) TSS/TA, while the lowest (12%) was found in the case of the B+SMAP treatment. A similar trend in TSS/acidity was observed when the blackberries were stored at 30 °C for 3 days (Figure 6).

The findings of our study regarding TSS/TA are consistent with the observations of Gupta et al. [70], who reported that the TSS/acid ratio of peaches gradually increased throughout their storage span. This could be associated with the progressive rise in the overall concentration of soluble solids and the corresponding decrease in fruit acidity over time.

### 2.7. Effect of Sonicated Edible Coating Gel on Total Flavonoid Content (TFC)

**Figure 7:**
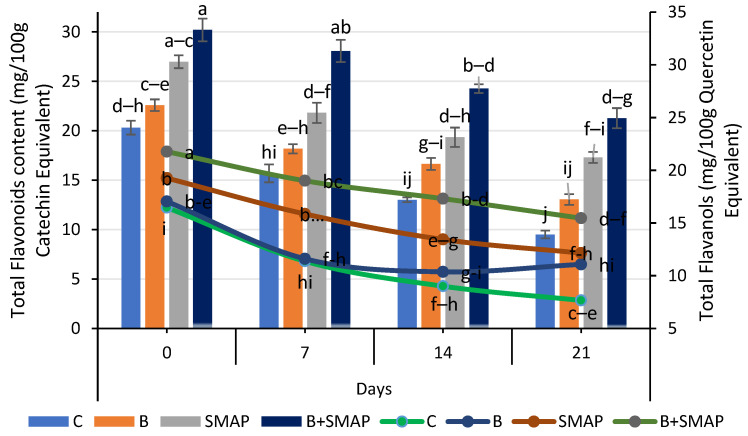
Total flavonoid content (mg/100 g catechin equivalent) and total flavanols (mg/100 g quercetin equivalent) of different treatments of blackberries stored at 4 °C. Total flavonoid content is shown on primary vertical axis (bars) and total flavanols on secondary vertical axis (line). C (control without coating gel), B (blanched), SMAP (coated with SMAP coating gel), B+SMAP (blanched and coated with SMAP coating gel). Different letters show significant differences between treatments at *p* ≤ 0.050.

**Figure 8:**
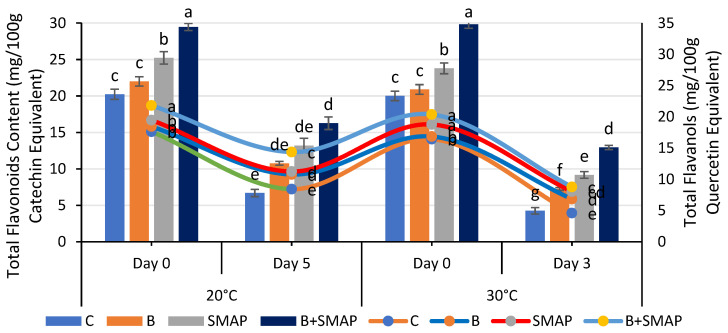
Total flavonoid content (mg/100 g catechin equivalent) and total flavanols (mg/100 g quercetin equivalent) of different treatments of blackberries stored at 20 and 30 °C. Total flavonoid content is shown on primary vertical axis (bars) and total flavanols on secondary vertical axis (line). C (control without coating gel), B (blanched), SMAP (coated with SMAP coating gel), B+SMAP (blanched and coated with SMAP coating gel). Different letters show significant differences between treatments at *p* ≤ 0.050.

Blackberry fruits showed a reduction in TFC in response to different storage temperatures and intervals, but this reduction was smaller under the synergistic effect of B and the SMAP edible coating gel. The B+SMAP-treated fruits stored at 4 °C for 21 days maintained the highest TFC compared to the control fruits. The B+SMAP treatment reduced the TFC loss by 24% relative to the uncoated control fruits (Figure 7). At 20 °C, after 5 days, the B+SMAP-treated fruits again exhibited a higher TFC than the controls. Similarly, at 30 °C, after 3 days, the B+SMAP-treated fruits exhibited the highest TFC, demonstrating the treatment’s greater effectiveness (Figure 8).

The application of the SMAP edible coating gels in combination with fruit blanching proved to be the most efficient formulation for inhibiting the degradation of TFC during storage. The findings of our study aligned with those of Khalifa et al. [71], who observed that the application of a chitosan olive-oil-based coating on strawberries mitigated the reduction in TFC. According to the findings of Riaz et al. [72], it was observed that uncoated fruits exhibited notably lower levels of flavonoids in comparison to fruits coated with chitosan-based apple peel polyphenols. Likewise, the flavonoid content was reduced in thornless blackberries kept at an elevated temperature (25 °C), regardless of the type of packaging materials used [73]. Our findings regarding the TFC agree with previous reports on blackberries, confirming a smaller reduction in the TFC in response to fruit coating [74].

### 2.8. Effect of Sonicated Edible Coating Gel on Total Flavanols (TFls)

A consistent decrease in the TFls was observed in both treated and non-treated fruits when stored at different temperatures for specific intervals. Among the treatments, the B+SMAP-treated fruits stored at 4 °C for 21 days and at 20 °C for 5 days showed the lowest reduction in TFls of 24% and 18%, respectively, compared to the untreated fruits (Figure 7). Similarly, at 30 °C after 3 days, the B+SMAP treatment inhibited the degradation of TFls by 15% compared to the control treatment (Figure 8).

### 2.9. Effect of Sonicated Edible Coating Gel on Total Phenolic Content (TPC)

**Figure 9:**
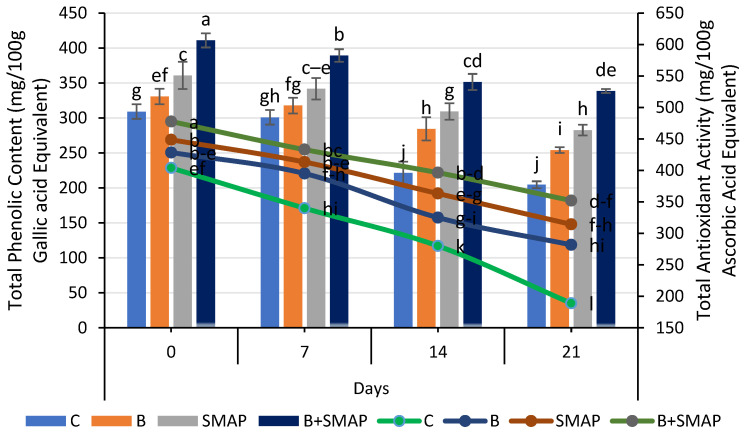
Total phenolic content (mg/100 g gallic acid equivalent) and total antioxidant activity (mg/100 g ascorbic acid equivalent) of different treatments of blackberries stored at 4 °C. Total phenolic content is shown on primary vertical axis (bars) and total antioxidant activity on secondary vertical axis (line). C (control without coating gel), B (blanched), SMAP (coated with SMAP coating gel), B+SMAP (blanched and coated with SMAP coating gel). Different letters show significant differences between treatments at *p* ≤ 0.050.

**Figure 10:**
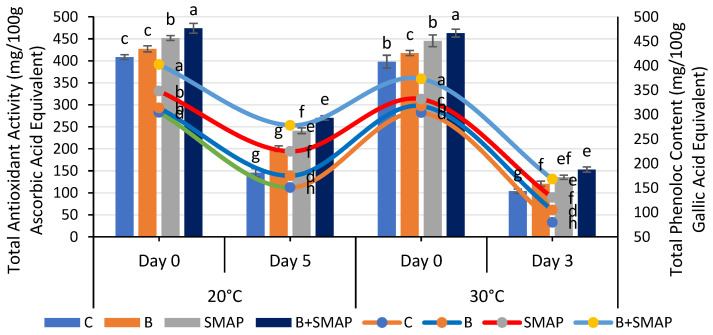
Total antioxidant activity (mg/100 g ascorbic acid equivalent) and total phenolic content (mg/100 g gallic acid equivalent) of different treatments of blackberries stored at 20 and 30 °C. Total antioxidant activity is shown on primary vertical axis (bars) and total phenolic content on secondary vertical axis (line). C (control without coating gel), B (blanched), SMAP (coated with SMAP coating gel), B+SMAP (blanched and coated with SMAP coating gel). Different letters show significant differences between treatments at *p* ≤ 0.050.

The total phenolic content (TPC) of the blackberries decreased at various storage temperatures (4, 20 and 30 °C). At 4 °C, after 21 days, the B+SMAP-treated fruits exhibited the highest TPC compared to the control. The B+SMAP treatment retained 16%, 30% and 18% more TPC when stored at 4, 20 and 30 °C for 21, 5 and 3 days, respectively, than the control fruits (Figure 9 and Figure 10).

The blackberry fruits showed a decline in the TPC, but the sonicated edible coating gel proved to be an efficient formulation and demonstrated a considerable ability to inhibit the loss of total phenolic content during storage. A reduction in the TPC throughout storage could be attributed to the potential disintegration of the cell structure resulting in fruit leakage and decay [61,75,76,77]. Various reports depicted the effect of edible coatings in maintaining a higher TPC in blackberries [57,61,78].

### 2.10. Effect of Sonicated Edible Coating Gel on Total Antioxidant Activity

A gradual loss in the total antioxidant activity was observed in all of the treatments stored at 4, 20 and 30 °C as the storage time progressed, regardless of whether the fruits were treated or untreated. At 4 °C, the B+SMAP-treated fruits showed a considerable retention of total antioxidant activity, i.e., about a 27% lower reduction in total antioxidant activity after 21 days compared to the untreated fruits (Figure 9). Likewise, at 20 and 30 °C, the B+SMAP-treated fruits showed a 21% and 7% lower decline in antioxidant activity, respectively, compared to the control (Figure 10).

A decline in the total antioxidant activity, probably associated with fruit senescence, leads to a reduction in anthocyanin production [79]. From our findings, it can be inferred that the application of the SMAP edible coating effectively enhanced the antioxidant activity in blackberry fruits. The findings of our results are consistent with those of De Bruno et al. [56], who found that edible coatings enriched with bergamot essential oil improved antioxidant activity in strawberries under storage conditions. Numerous studies demonstrated the efficacy of edible coatings in effectively preserving the antioxidant content of both whole fruit (blackberries) and in freshly cut slices of apple, kiwifruit and papaya [80,81]. Antioxidant activity in blackberries is associated with phenolic compounds and organic acids. The loss of these compounds during storage is mainly responsible for a decrease in the antioxidant activity [77]. The higher TPC in fruits treated with the SMAP edible coating could be attributed to high levels of antioxidant activity.

### 2.11. Effect of Sonicated Edible Coating Gel on DPPH Radical Scavenging Activity

**Figure 11:**
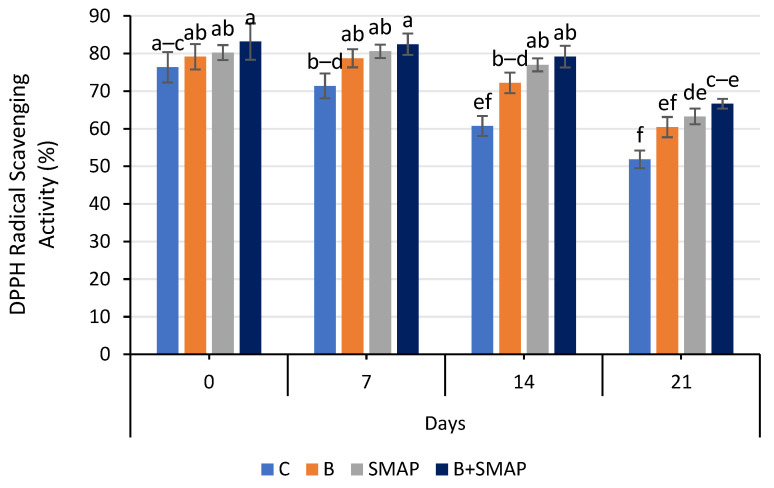
DPPH radical scavenging activity (%) of different treatments of blackberries stored at 4 °C. C (control without coating gel), B (blanched), SMAP (coated with SMAP coating gel), B+SMAP (blanched and coated with SMAP coating gel). Different alphabets show significant difference among treatments at *p* ≤ 0.050.

**Figure 12:**
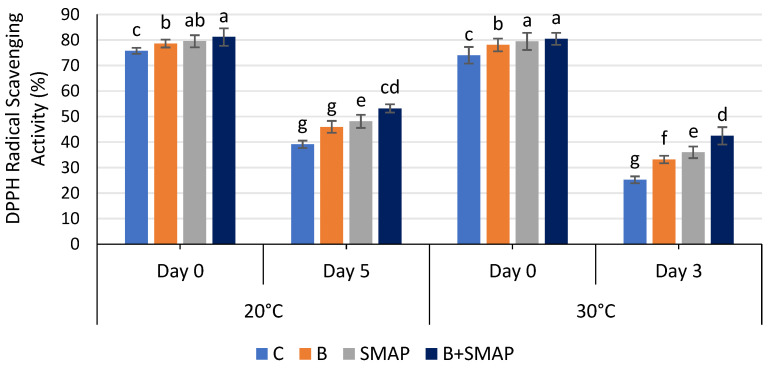
DPPH radical scavenging activity (%) of different treatments of blackberries stored at 20 and 30 °C. C (control without coating gel), B (blanched), SMAP (coated with SMAP coating gel), B+SMAP (blanched and coated with SMAP coating gel). Different alphbetes show significant difference among treatments at *p* ≤ 0.050.

The DPPH radical scavenging activity of blackberry fruits decreased gradually during storage at 4, 20 and 30 °C in all of the treated and untreated fruits. At 4 °C, fruit treated with B+SMAP showed a considerable retention in DPPH radical scavenging activity (12%) compared to the uncoated fruits after 21 days (Figure 11). All of the treatments stored at 20 and 30 °C showed a loss in DPPH radical scavenging activity. However, the B+SMAP-treated fruit displayed a 19% and 18% lower loss of DPPH radical scavenging activity, respectively, compared to the control treatment (Figure 12).

DPPH radical scavenging assays are employed to determine the efficacy of a chemical compound in combating free radicals. The ability to inhibit DPPH radicals improves with rising essential oil concentrations [82]. The results of this study demonstrate the effectiveness of blanching and the SMAP coating gel in preserving the DPPH radical scavenging activity of blackberries during storage. The combination of blanching and the SMAP coating gel can help retain the antioxidant activity of blackberries at 4 °C. The coating gel may have acted as a barrier, preventing moisture and oxygen from penetrating the fruit and causing degradation of the antioxidants. Blanching inactivates the polyphenol oxidase enzyme, which causes a decrease in polyphenol and flavonoid content, resulting in decreased DPPH radical scavenging activity [83]. Our findings are consistent with previous studies that have confirmed the benefits of blanching and coating in preserving the antioxidant activity of fruits. For example, blanching and coating with an edible coating gel of starch and beeswax helped preserve the antioxidant activity of papaya during storage [84]. Furthermore, pectin and CMC-based edible coatings were proven to be effective at preserving the DPPH radical scavenging activity of plum fruit and blueberries, respectively, during storage [85,86].

## 3. Conclusions

The application of a sonicated and microwave-assisted pasteurized (SMAP) edible coating gel containing orange peel essential oil on blackberry fruit showed considerable effects on its quality attributes, especially at a refrigerated temperature. These emulsions successfully reduced decay and weight loss, thereby retained firmness and higher antioxidant activity in terms of total phenolics, flavonoids and flavanols. Similarly, the SMAP gel in combination with blanching managed to slow down mycelium growth and fruit quality deterioration. It may be concluded that CMC-based sonicated edible coating gels incorporated with natural orange peel oil could have maintained the postharvest quality of blackberries during refrigerated storage. However, further research is required to explore the potential of sonicated edible coating gels with various essential oils on different horticultural commodities across diverse storage conditions.

## 4. Materials and Methods

### 4.1. Materials

Blackberries (cv. Osage) collected from the local market were sorted and washed with distilled water. After washing, half of the fruits were blanched in distilled water at 70 °C for 3 min [87]. After blanching, fruits were cooled rapidly to 4 °C. NaOH, phenolphthalein, DPPH, Folin–Ciocalteu reagent, ethanol, gallic acid, ascorbic acid, Na_2_CO_3_, sodium phosphate (Na_2_HPO_4_·12H_2_O), H_2_SO_4_, ammonium molybdate, catechin, quercetin, sodium nitrite and aluminum chloride were purchased from Sigma Aldrich, Saint Louis, MO, USA. All standards and chemicals used were of analytical grade. Citrus essential oil was purchased from the manufacturer Nature’s Oil.

### 4.2. Development of Sonicated and MAP Coating Gel

The coating gel was prepared by dissolving 0.5% carboxymethyl cellulose (*w*/*v*) in distilled water, along with 0.75% glycerol (1, 2, 3-Propanetriol, C_3_H_8_O_3_) as a plasticizer. Then, 2 mL of Polysorbate 80 (polyoxyethylene-20 sorbitan-monooleate, C_64_H_124_O_26_), also known as Tween-80, was added as a non-ionic surfactant to the emulsion. After adding citrus peel essential oil (0.5%), the whole mixture of emulsion was continuously agitated using blender (Waring MX1050XTS Xtreme, McConnellsburg, PA, USA) at 30,000 rpm for 5 min. In order to reduce droplet size, the resultant gel was ultrasonicated using probe of 15 mm diameter at a frequency of 20 kHz and power of 950 Watts (CGOLDENWALL Sonicator processor; model 13MPZ24082203, Made in China, available at Glendale, California 91201, USA) for 10 min on a pulse mode with an interval of 5 sec on and 5 sec off. The sonicated emulsion was processed for microwave (Model No: DW-131A) treatment in the following conditions: frequency of 2450 MHz, power of 1000 Watts, temperature of 90 °C and duration of 60 s.

### 4.3. Treatment Plan

Blackberries were divided into four groups, with each group consisting of 100 fully mature and uniformly sized fruits. Analysis was performed in triplicate for each treatment. Fruits were sorted and washed with distilled water. Afterwards, the fruits were given the following treatments: C (control without coating gel), B (blanching), SMAP (sonicated and microwave-assisted pasteurized edible coating gel), B+SMAP (blanching and SMAP coating gel). All treated and control fruits were kept at 4, 20 and 30 °C for 21, 5 and 3 days for different fruit quality analyses.

#### Economic Feasibility Analysis

The cost of preparation for the coating gel (Table 1) indicated that it is very economical and only approximately 300 mL of gel was used to coat 5 kg of blackberries. Further, sonication, microwave processing and ingredients of gels had no negative impact on taste and texture of fruits; in fact, citrus peel oil contributed to the fruits’ pleasant taste. Sonication process facilitated the homogenous mixing of all ingredients and microwaving increased the viscosity of the gel.

### 4.4. Postharvest Quality Attributes

Twenty-five fruits per replicate (3 replicates) were used for weight loss (%) determination. Weight loss was calculated by weighing fruits at each storage interval. Results are expressed as a percentage of weight loss.

(1) $$Weight\;loss\;(\%)=\frac{\left(Initial\;weight-Final\;weight\right)}{Initial\;weight}\times 100$$

Viscosity and antimicrobial and sensory properties of the coating gel are very important parameters that may impact the fruits’ protection. The developed coating gel was not thick enough to incite anaerobic respiration. The essential oil used possesses excellent antimicrobial properties. Moreover, the microwave-assisted pasteurization of gel further insured the fruits’ microbial safety. The amount of deteriorated fruit in each treatment was assessed at storage intervals up to 21 days. The decay percentage of fruit for each treatment was determined by using the following formula:

(2) $$Decay\;(\%)=\frac{Number\;of\;decayed\;fruits}{Number\;of\;total\;fruits}\times 100$$

About 10 g of blackberry flesh was homogenized with distilled water (100 mL) and centrifuged (Jouan C312 benchtop centrifuge, Saint Herblain, France) at 4500 rpm for 30 min, followed by filtration through Whatman #1 filter paper. Its pH was determined with a pH meter (HI9124, Hanna Instruments, Smithfield, RI, USA). Titratable acidity was determined by titrating filtrate with 0.1 M NaOH solution and adding phenolphthalein (0.5 mL) as an indicator. Titratable acidity was expressed as % citric acid per 100 g fresh weight. About 10 g of blackberry fruit pulp was homogenized, centrifuged and filtered. Total soluble solids expressed as °Brix were determined using a refractometer (Atago Co., Tokyo, Japan). The ripeness, also known as the Maturity Index, of fruit was calculated by dividing its TSS by TA, as previously carried out by Rahman et al. [88].

(3) $$MI=\frac{Brix\;(TSS)}{titratable\;acidity\;(TA)}$$

Total phenolic content was determined using Folin–Ciocalteu reagent with slight modifications, as previously carried out by Qureshi et al. [89], and sample absorbance was measured using a 96-well microplate reader (Agilent Biotek Synergy Neo2, Santa Clara, CA, USA) at 760 nm. Standard solutions of gallic acid in ethanol were also employed. Total phenols were represented in terms of gallic acid equivalent (GAE) per 100 g.

Total flavonoid content was determined by the method explained by Qureshi et al. [89]. The sample’s absorbance was noted at a wavelength of 510 nm using a 96-well microplate reader. Standard solutions of catechin were also used to generate a curve and R^2^ equation. The results were expressed in milligrams of (+)-catechin equivalent (CE) per 100 g of weight.

Total antioxidant activity was measured using a 96-well microplate reader at 695 nm [90]. The findings are presented in the form of micrograms of (+)-ascorbic acid equivalent (AAE) per 100 g of weight, with ascorbic acid serving as a standard.

The DPPH radical scavenging activity (RSA) was determined by following the standard procedure. A total of 0.01 g DPPH was dissolved in 125 mL ethanol. Absorbance reading at 517 nm was taken using a 96-well microplate reader [90]. DPPH RSA of samples was measured as (%) using the following equation:

(4) $$DPPH\;RSA\;(\%)=\frac{Control\;absorbance-absorbance\;of\;sample}{Control\;absorbance}\times 100$$

### 4.5. Statistical Analysis

The results were subjected to two-way analysis of variance (ANOVA) using Minitab 16 software, and Tukey’s (HSD) test was used to describe the means with 95% (*p* < 0.05) confidence.

## Figures and Tables

**Table 1:** Cost estimation for preparation of 1L sonicated coating gel.

Sr No	Ingredient	Quantity Used	Price per Package ($)	Cost for 1 L ($)
1	Carboxymethyl cellulose	5 g/L	21.20/Lb	0.23
2	Glycerol	7.5 mL	157/500 mL	2.36
3	Tween-80	2 mL	87.40/500 mL	0.35
4	Citrus peel essential oil	5 mL/L	22.20/500 mL	0.22
5	Distilled water	1 L	1.37/gal	0.36
6	Overhead charges			0.70
	Total cost for making 1 L coating gel			4.22

## Data Availability

All data are available within the manuscript.

## Abbreviations

The following abbreviations are used in this manuscript:

DPPH	2,2-diphenyl-1-picrylhydrazyl
S	Sonicated
MAP	Microwave-assisted pasteurized
SMAP	Sonicated and microwave-assisted pasteurized
CPEO	Citrus peel essential oil
C	Control
B	Blanched
B+SMAP	Blanched + coated
CMC	Carboxy methyl cellulose
GAE	Gallic acid equivalent
CE	Catechin equivalent
AAE	Ascorbic acid equivalent
RSA	Radical scavenging activity
ANOVA	Analysis of variance
HSD	Honestly significant difference
p	Probability
TSS	Total soluble solids
TA	Titratable acidity
TFC	Total flavonoid content
TPC	Total phenolic content
TFls	Total flavanols
GRAS	Generally recognized as safe
USFDA	United States Food and Drug Administration
